# Managing health research capacity strengthening consortia: a systematised review of the published literature

**DOI:** 10.1136/bmjgh-2018-001318

**Published:** 2019-04-14

**Authors:** Nadia Tagoe, Sassy Molyneux, Justin Pulford, Violet I Murunga, Sam Kinyanjui

**Affiliations:** 1 KEMRI Wellcome Trust Research Programme, Kilifi, Kenya; 2 Office of Grants and Research, Kwame Nkrumah University of Science and Technology, Kumasi, Ghana; 3 Nuffield Department of Medicine, Centre for Tropical Medicine and Global Health, University of Oxford, Oxford, UK; 4 Department of International Public Health, Centre for Capacity Research, Liverpool School of Tropical Medicine, Liverpool, UK; 5 Faculty of Health and Life Sciences, University of Liverpool, Liverpool, UK

**Keywords:** health, research, capacity strengthening, consortium, management

## Abstract

**Background:**

Locally relevant research is considered critical for advancing health and development in low- and middle-income countries (LMICs). Accordingly, health research capacity strengthening (HRCS) efforts have intensified, increasingly through consortia. Yet, the knowledge base for managing such consortia is not well defined. This review aimed to ascertain the scope and quality of published literature on HRCS consortium management processes, management-related factors influencing consortium operations and outcomes, and the knowledge gaps.

**Methods:**

Given the paucity of published HRCS literature, a ‘systematised review’ as outlined by Grant and Booth was conducted, modelling the systematic review process without restriction to research-based publications. A systematic search in PubMed and Scopus was carried out coupled with a manual search for papers using reference checking and citation searching. A quality appraisal of eligible articles using the Mixed Method Appraisal Tool was undertaken. Thematic synthesis was used to analyse the extracted data.

**Results:**

The search identified 55 papers, made up of 18 empirical papers and 37 commentaries focusing on consortium-based HRCS initiatives involving LMICs and reporting management-related data. The review indicates increasing efforts being made in the HRCS field in reporting consortia outcomes. However, it highlights the dearth of high-quality empirical research on HRCS consortium management and the nascent nature of the field with most papers published after 2010. The available literature highlights the importance of relational management factors such as equity and power relations in influencing consortium success, though these factors were not explored in depth. Operational management processes and their role in the capacity strengthening pathway were rarely examined.

**Conclusion:**

Findings indicate a weak evidence base for HRCS consortium management both in terms of quantity and conceptual depth, demonstrating the need for an expanded research effort to inform HRCS practice.

**Key questions**

**What is already known?**

- The recognition of the fundamental role of research in advancing health and development has resulted in substantial investments in health research capacity strengthening (HRCS) consortia in low- and middle-income country settings.

**What are the new findings?**

- Very little attention has been given to consortium management in the literature, and the current evidence is characterised by a lack of high-quality empirical research.
- The available evidence highlights the importance of relational elements of consortium management such as equity and power relations but does not explore these elements in depth. Operational management processes adopted and their role in the capacity strengthening pathway were rarely examined.

**What do the new findings imply?**

- There is a need to strengthen the evidence base on the role and contribution of consortium management processes to broader HRCS capacity development initiatives.

## Introduction

Health research has been recognised as an essential tool in addressing health and development challenges, yet the capacity of many low- and middle-income countries (LMICs) to conduct locally relevant research is still low.^1 2^ In the last three decades, several calls to action have been made for sustainable health research capacity strengthening (HRCS) in LMICs,^1–4^ resulting in substantial investments in a wide range of initiatives.^5–7^ Mechanisms for developing research capacity in LMICs have evolved over the years, progressing from the provision of technical assistance to individual-focused training, and more recently towards institutional and system-wide approaches.^8 9^ One of the main strategies adopted over the period has been the teaming up of institutions to implement these programmes.^6 10^ Though such groupings refer to themselves by various names such as partnership, consortium, and network,^11–13^ we will use the term ‘consortium’ in this paper.

HRCS consortia typically consist of individuals and institutions from both high- and low- and middle-income countries pooling their varying levels of resources, expertise and experience and working together towards collective gains in health research capacity.^14 15^ While these consortia are often led by high-income country partners,^16 17^ there is a rising trend of LMIC-led consortia such as those that were supported by the Wellcome Trust’s African Institutions Initiative and its successor, the Developing Excellence in Leadership, Training and Science Africa Initiative, the USA National Institute of Health’s Medical Education Partnership Initiative, and the European and Developing Countries Clinical Trials Partnership Programmes.

The increase in HRCS consortia has heightened the need to assess their activities and effectiveness. Accounts of HRCS consortia in the literature have generally focused on programme activities and outputs and associated successes and challenges.^18–20^ However, current evaluation thinking embraces the value of processes in addition to outcomes,^21 22^ recognising that assessing programme implementation processes to determine how and why specific outputs are realised is as important as assessing the outputs themselves.^22 23^ Integral to programme implementation processes are the management structures and activities employed throughout its lifecycle.^24^ Managing a consortium is often a complex effort involving coordination of both activities and partners (individual and institutional) that are, in turn, embedded in additional structures and systems.^25 26^ Leaders of multimillion dollar HRCS consortia, who are often primarily researchers, are expected to deal with these managerial complexities.^25^ The evidence base to support the navigation of this complex endeavour in the HRCS context is neither well defined nor sufficiently understood.^27–29^

There are indicators of increasing attention to consortium management practices in HRCS initiatives. Examples include the requirement by some funding bodies for explicitly stated consortium management outputs in programme theories of change,^30^ and the development of consortium management tools such as the research fairness initiative^31^ and guides for research partnerships.^32 33^ It is clear that consortium management is an integral part of the global HRCS effort, and a robust evidence base including understanding consortium management processes and practices and their effectiveness is essential. This review aims to ascertain the breadth, depth and quality of the published evidence on HRCS consortium management, and identify the management processes, experiences and key issues raised by consortium actors, and the knowledge gaps in the available evidence.

## Methods

### Type of review

Due to the paucity of robust HRCS research publications,^34^ conducting a standardised systematic review which requires high-quality research evidence^35 36^ was not feasible. We thus conducted a systematised review, which models the systematic review process without strict adherence to study inclusion criteria.^35^ We aimed to be widely inclusive to draw out the full range of HRCS consortium management-related data in the published literature, necessitating the inclusion of all types of peer-reviewed literature without limitation to publication type (research based or not) and quality.

### Data sources, search strategy and selection of papers

A systematic electronic search of peer-reviewed articles using PubMed and Scopus was conducted without any date restrictions. The search was limited to peer-reviewed literature as the aim of this review is to identify the extent of and findings from existing scientific literature pertaining to HRCS consortium management. The search terms used were (1) health AND (2) research AND (3) capacity AND (4) strengthening AND (5) consortium AND (6) LMICs, together with variants of some of the terms (online supplementary table S1). LMIC is defined according to the current World Bank classifications.^37^ Four geographical regions with the highest concentration of LMICs namely Africa, Asia, Latin America and the Caribbean, and Pacific were included to optimise the search. Results were saved in an Endnote X8 library.

10.1136/bmjgh-2018-001318.supp1 Supplementary data

Identified papers were first screened by the first author against the inclusion criteria using titles and abstract. An article was included if it (1) focused on one or more consortium-based HRCS initiatives; (2) involved LMICs and (3) included descriptions, processes, findings or reflections related to the establishment and ongoing management of consortia. Additional criteria were papers published up to December 2018 with both abstract and full paper available in English. The restriction to include only papers written in English was due to lack of resources for translation and time limitations. Articles were retained for full-text review if they met the criteria or more information was required to decide, after which the final selection was made. There was an agreed process for team consultations when it was unclear whether or not to include a paper. Additional papers were identified by a manual search which included checking the references and supplementary lists of identified articles and citation searching.^38^

### Quality appraisal

Though there was no quality threshold for inclusion, an appraisal of the selected articles was carried out to give an indication of the quality of the current evidence on HRCS consortium management. The Mixed Methods Appraisal Tool (MMAT) was used due to its suitability for appraising multiple design studies.^39^ The tool includes screening questions which assess the eligibility of papers for full appraisal. It comprises sets of criteria for qualitative, quantitative and mixed studies, and metrics for determining the overall quality score for each study.^40^ The empirical papers were screened and the qualifying papers assessed for methodological quality and scored. A second reviewer conducted an independent appraisal of all the papers. An initial discussion between the two reviewers was held in advance to ensure a common understanding of the tool. A third reviewer facilitated the resolution of any divergences.

### Data extraction and analysis

Data were extracted from the selected papers using the matrix method.^41^ This method provides a structured way of recording extracted information and findings from each publication using a table, facilitating a systematic synthesis process. Columns representing specific areas of interest were used to capture data. These included the following: publication authors and year; characteristics of the HRCS programme such as goals, main activities and geographical focus; and consortium characteristics such as structure, size and composition. Study objectives and design, methods used in data collection, sampling and analysis, and frameworks or guidelines applied were also obtained from empirical papers. Findings from each paper were categorised into three broad areas: (1) descriptions of management processes and systems adopted during the consortium’s formation and implementation, (2) experiences and lessons learnt and (3) effect of the processes and experiences on the achievement of programme goals. A thematic synthesis of the extracted data was then carried out which involved inductively identifying any descriptive and analytical themes, as well as similarities, divergences and associations across papers. To strengthen the rigour of the process, each step and output was independently assessed by a second reviewer.

## Results

### Study selection

**Figure 1:**
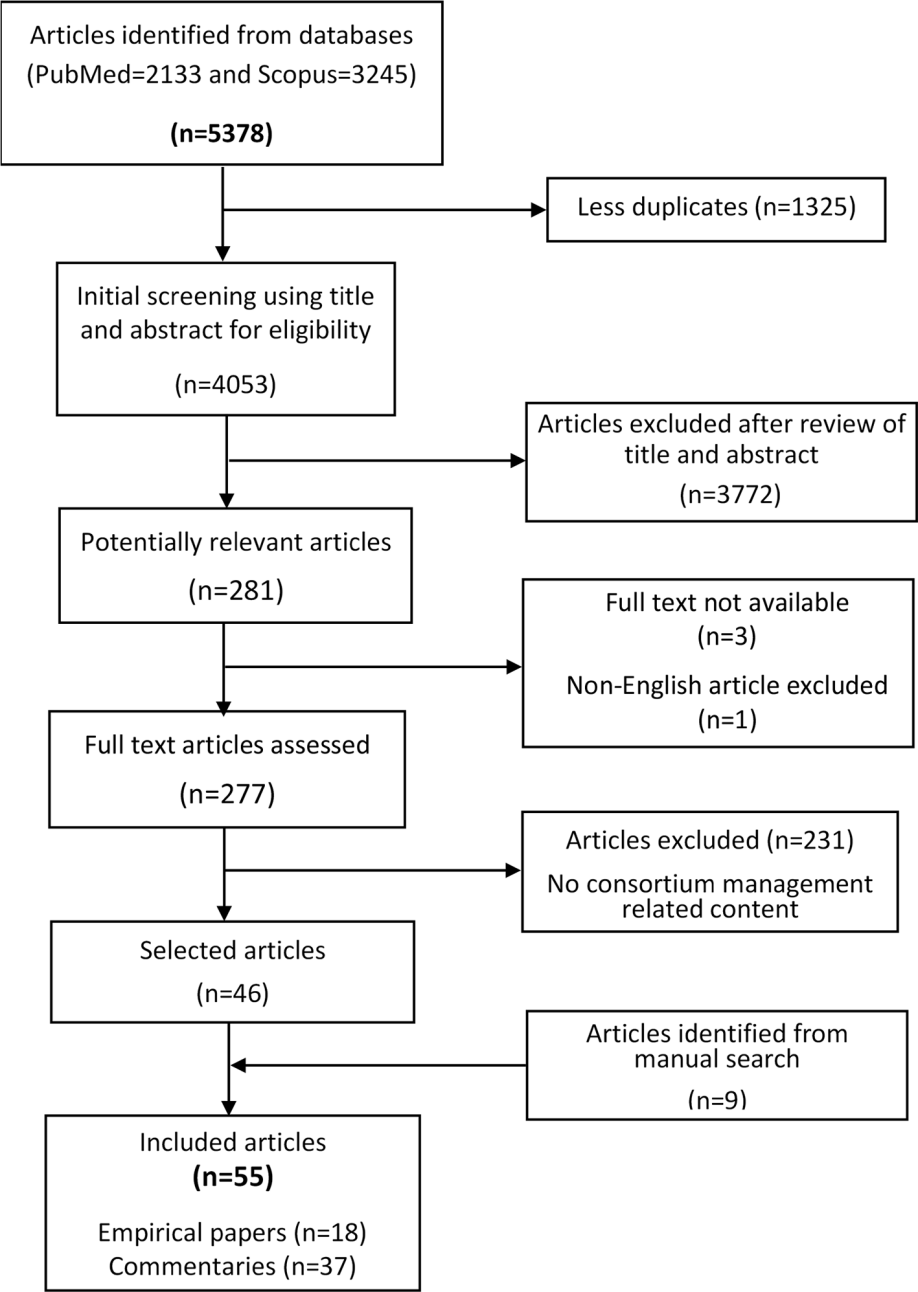
Paper screening and selection process.

The electronic search yielded 5378 papers of which 1325 duplicates were removed, retaining 4053 (figure 1). In all, 3772 articles were rejected based on a review of the title and abstract, and an additional four were excluded as the full texts were not accessible for three and the fourth was not available in English. Of the 277 potentially relevant articles, 46 were retained after a full-text review, and a manual search identified nine additional articles, resulting in 55 included papers made up of 18 empirical papers and 37 commentaries (table 1). A detailed summary of the papers is presented in online supplementary table S2 and S3.

**Table 1:** Summary of publication and programme characteristics

Category	Characteristic	Description	No. and percentage of publications
Publication characteristics (N=55)	Type of publication	Empirical research	18 (33%)
		Commentary	37 (67%)
	First author affiliation	HIC	36 (66%)
		U-MIC	4 (7%)
		L-MIC	6 (11%)
		LIC	9 (16%)
	Last author affiliation	HIC	38 (69%)
		U-MIC	10 (18%)
		L-MIC	3 (6%)
		LIC	4 (7%)
Programme/ consortium characteristics (N=51)	Geographical focus^*^	Africa	37 (73%)
		Asia	12 (24%)
		Latin America and the Caribbean	7 (14%)
		Pacific	1 (2%)
	Consortium leadership	HIC	32 (63%)
		U-MIC	2 (4%)
		L-MIC	3 (6%)
		LIC	2 (4%)
		Led by both L-MIC and LIC partners	2 (4%)
		Not indicated	10 (19%)
	Capacity strengthening focus	Dedicated RCS initiatives	23 (45%)
		Embedded RCS initiatives	28 (55%)
	Subject focus	Disease or discipline focus	38 (74%)
		Generic	11 (22%)
		Not indicated	2 (4%)
	Main activities*	Training individuals	40 (78%)
		Collaborative research	25 (49%)
		Institutional capacity enhancement	11 (22%)
		Developing collaborations	9 (18%)
		Knowledge translation	9 (18%)
		Infrastructure enhancement	4 (8%)

*Some programmes combined two or more categories.

HIC, high-income country; LIC, low-income country; L-MIC, lower middle-income country; RCS, research capacity strengthening; U-MIC, upper middle-income country.

### Characteristics of included papers

**Figure 2:**
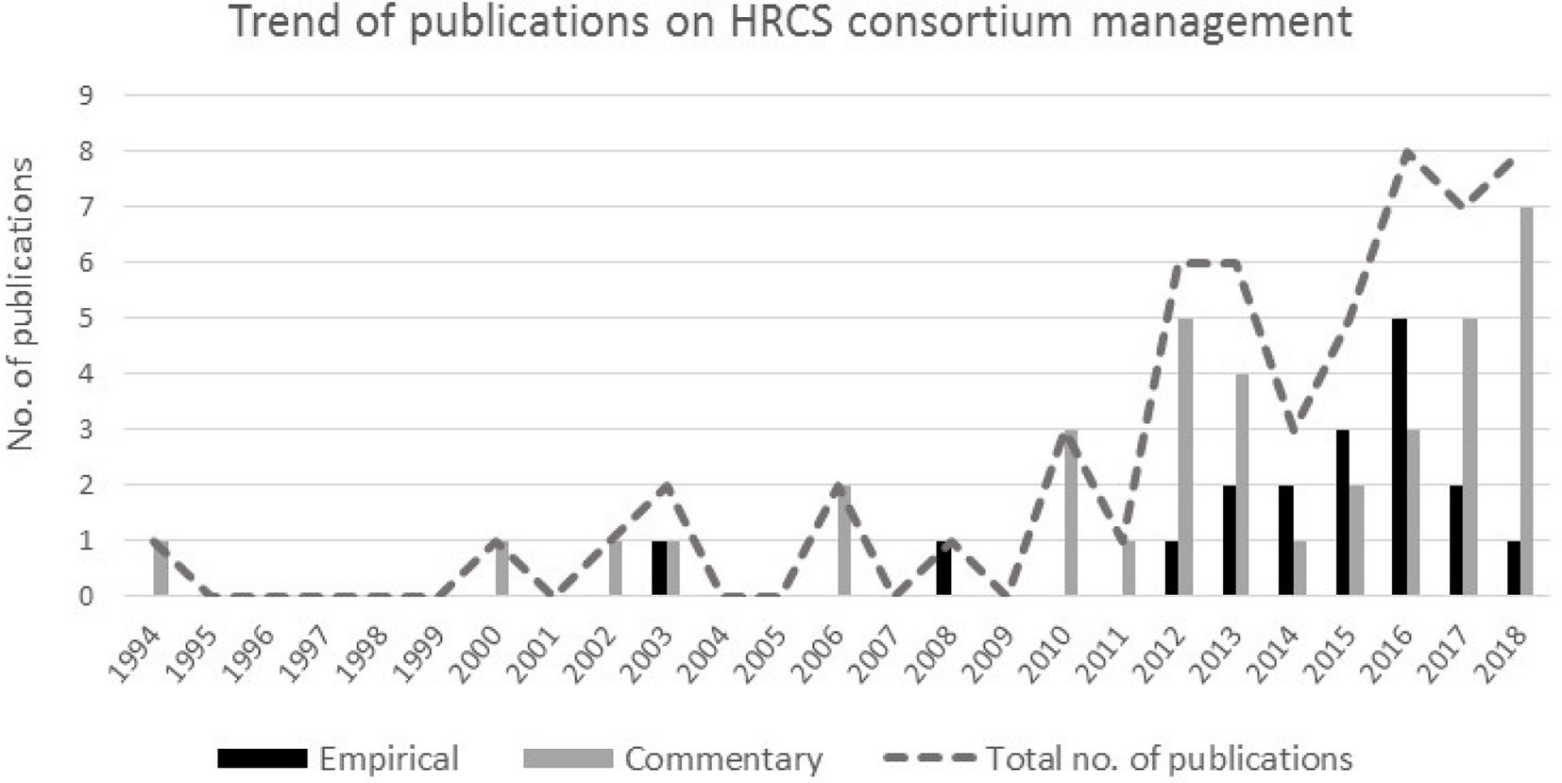
Number and type of publications per year. HRCS, health research capacity strengthening.

Only one paper was published before 2000, with the majority (47 out of 55) published between 2010 and 2017, indicating a sixfold increase compared with the period preceding 2010 (figure 2). The highest number of papers published in a year was eight. Half of the papers (n=28) had neither the first nor last authors affiliated to LMIC institutions, and in a fifth, there were no LMIC-affiliated authors at all (table 1). Last authors (48 out of 55) were primarily affiliated to high- and upper middle-income countries.

There were twice as many commentaries as empirical research papers (table 1), with 14/18 empirical papers based on qualitative studies and four on mixed methods. Almost all empirical papers (n=17) had a learning and evaluation focus, 10 of which were conducted internally and the rest by external assessors. Only seven qualitative papers were scored in the top half of the MMAT quality range (online supplementary table S2) based on having clear research objectives, using data sources and analysis approaches relevant for addressing the research questions, and giving appropriate consideration to how the findings relate to the context while the other qualitative research papers presented very little data on these. The remaining papers had used mixed-method approaches without clearly indicating the rationale or data integration process or adhering to sampling and other quantitative methodological criteria.^40^

Included papers sought to evaluate or reflect on the consortium’s operations particularly on the activities and outputs, with only a third primarily focusing on the partnership experience, assessing the successes, challenges and lessons learnt from the perspective of consortium actors. Evaluation of consortium management processes was the sole or prominent aim of only two papers,^42 43^ one of which happened to be the only paper reporting a failed consortium.

### Description of consortia

The 55 identified papers represented 51 distinct HRCS programmes, as three programmes were reported in several publications. There was an inconsistent use of terms in describing the collaborative set-ups, with 39 papers using two or more terms interchangeably, and one paper using five. The most commonly used terms were partnership (n=22), network (n=11) and consortium (n=10), and fewer uses of collaboration (n=4), alliance (n=2) and community of practice (n=2). Only five papers provided definitions of the used terms, which varied considerably.^44–48^

The 51 consortia varied widely in size, ranging from 2 to 20 institutional partners. The HRCS programmes included LMICs, mostly in Africa (n=38). Of the 41 consortia that had reported on leadership, 32 were led by high-income country partners (table 1). As shown in table 1, HRCS was either the primary focus of the programmes or a component of a broader research, educational or clinical care programme. Consortia sought to build capacity using a single or combination of activities, mostly training of individuals (short term and degree awarding) and learning ‘on the job’ through conducting collaborative research. None of the papers indicated the process used or factors that determined the selection of HRCS activities.

### Operational aspects of consortium management

A range of management structures and processes adopted by consortia during their inception and implementation phases were reported across papers. These included partner selection and partnership development during the inception phase, and management structures, coordination, and monitoring during the implementation phase. These were neither the primary focus of the publications nor examined in detail, but rather brief descriptions introducing or providing context for studies and reflections. Below, we present data on operational processes used in the HRCS inception and implementation phases as well as data on relational aspects of consortium functioning (table 2).

**Table 2:** Summary of management issues raised across papers

Category	Description	No. of publications (%) (N=55)
Operational elements of management	Partner selection criteria	22 (40%)
	Determinants of consortium leaders	8 (17%)
	Partnership development phase	11 (20%)
	Types of collaborative agreement used	7 (13%)
	Governance structures	19 (35%)
	Coordination of consortia activities	21 (38%)
	Monitoring and evaluation of consortia activities	22 (40%)
Relational elements of management	Relationship building	45 (81%)
	Equity and power	24 (44%)
	Role of leadership	20 (36%)
	Partner inclusion	16 (29%)

#### Inception processes

**Figure 3:**
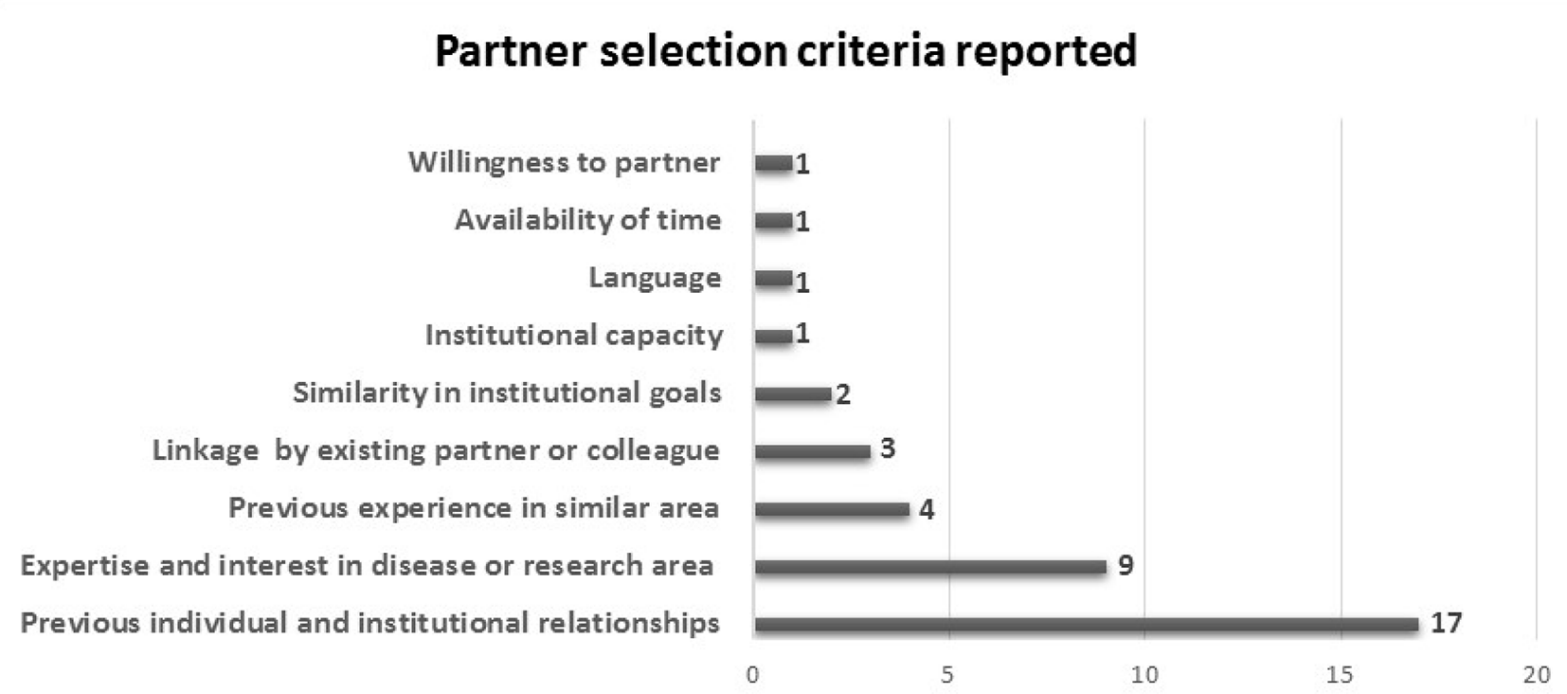
Partner selection criteria used and the number of publications that mention criteria.

Most consortia were formed in response to an HRCS funding opportunity and were initiated by the primary grant holder or principal applicant. There was one exception, where formation was the initiative of a government representative from the LMIC.^49^ Criteria for partner selection were discussed in 22 papers (figure 3), with the most cited criteria being previous individual and institutional working relationships (n=17), and expertise or experience in the disease or research area (n=9). Many papers (n=11) reported considering two or more criteria. It was not indicated in any paper if there were any considerations for determining the number or type of partners.

In 11 cases, consortia reported engaging in a partnership development process also referred to as an ‘engagement phase’,^50^ ‘inception phase’^42 51^ or ‘establishment process’.^52^ Typically, this process was used to facilitate partner and stakeholder engagement, identify partner needs and expectations, determine consortium goals, assign roles, establish governance structures, consortium guidelines and procedures, and develop a plan of action. This phase or process was reported to promote openness, trust and build team work,^52^ as well as help partners acknowledge and deal with any assumptions held.^53^ Only three papers^51 54 55^ described the use of a framework or tool to guide this process, citing the Partnership Assessment Tool, the four-dimensional Appreciative Inquiry Framework and the International Participatory Research Framework, respectively.

#### Implementation processes

The governance structures adopted by consortia were reported in a third of the papers. Governing bodies were similar across consortia and generally fell into four categories: advisory bodies that provided strategic advice,^56–58^ steering committees that made strategic and operational decisions,^43 58 59^ executive teams responsible for the day-to-day management^58 60 61^ and implementation teams that executed consortium activities.^57 62 63^ These governing bodies were often made up of representatives from partner institutions. However, neither the factors informing the choice of management structure nor the effectiveness of the structures were discussed in any paper. One paper reported considering gender balance,^59^ and three described the involvement of junior researchers (in one case stating the capacity strengthening intent of the decision).^25 62 64^ The leaders of the consortia tended to be those who initiated the collaboration, had the required resources or were selected to fulfil funder requirements.^46 56 65^ Researchers frequently took the lead management role in consortia. The role of a project manager or coordinator was reported in only two cases.^58 60^ One consortium employed trainees in management and administrative roles, and though this resulted in managerial capacity, it adversely affected their training progress due to the additional responsibilities.^42^ The consortium management capacities of leaders and managers were neither mentioned nor discussed, although two papers pointed out the value of both management and technical expertise in leading consortia.^25 49^

In all, 21 papers mentioned activity coordination processes, and 22 indicated the incorporation of monitoring and evaluation elements. The most cited platforms for coordinating activities and monitoring progress were consortium meetings, management meetings and partner visits, as well as telephone and electronic communication. Factors reported to foster coordination and monitoring included regular communication, jointly determined goals and processes, previous working relationships, and the use of codes of conduct and guidelines.^60 66 67^ Lack of clarity about roles and guidelines,^43 50^ and difficulties in organising meetings due to physical distances, time differences, conflicting partner priorities, and poor internet connectivity were reported as barriers particularly in large-sized consortia.^60 68 69^ Most of the 18 evaluations reported, whether internally or externally conducted, were programmatic in nature, focused on assessing training and research outputs, with only six reporting on partner relations and partnership successes and challenges. Frameworks used to guide these evaluations were reported in six cases. These included the Swiss Commission for Research Partnerships with Developing Countries’ Guide for Transboundary Research Partnerships,^19 65^ Mercer *et al*’s^70^ Guidelines for Assessing Participatory Research Projects,^50^ the Capacity WORKS Model,^43^ Kernaghan’s types of partnerships^48^ and the realist methodology approach.^49^ These frameworks are orientated towards examining research partnerships more broadly, with only the Capacity WORKS model^71^ tailored specifically to capacity development programmes.

### Relational aspects of consortium management

The four critical factors identified from the range of successes, challenges, enablers, barriers and lessons learnt shared across papers were relational in nature specifically building partner relationships, equity and power, leadership and inclusion. Although interrelated, we present the data shared on these key factors in turn, returning to the potential interplays in the discussion.

### Partner relationships

The most discussed factor reported as influencing consortium success was the importance of fostering strong relationships between partners, with nearly all papers (n=45) commenting on this. The value of informal networks and friendships among individual partners in consortium success was emphasised.^26 42 43 69 72^ In addition to their influence on the achievement of programme deliverables and consortium sustainability, effective relationships were in themselves seen as capacity outcomes.^26 45 60 62^

While these [courses and workshops] were the quantifiable outputs…, much of the experiences in capacity building are not measurable: these may focus on relationship dynamics, work and the learning experienced by the participants involved (p. 4)^60^

Many participants reported that new relationships developed during the project implementation were the most important outcomes (p. 5)^60^

Partner relationships were fostered by principles such as openness, trust, mutual respect, transparency, shared commitment and recognition^42 66 73 74^; and practices such as establishing guiding principles and norms, joint planning and implementation processes and regular communication.^49 59^ The importance of recognising and leveraging on differences in partner needs, strengths, interests, objectives, expectations, contexts and culture to nurture effective relationships was also noted.^49 74–76^ Almost half of the papers (n=21) reported encountering challenges when partner differences were not acknowledged and monitored.^26 29 49 68 73–76^ At the same time, the investment required (in time and other resources) and practical challenges of building relationships were recognised, particularly when partners were spread across continents.^29 42 50 63 69 77 78^ As demonstrated in one study, participants ‘found the process of establishing relationships and reaching consensus… laborious and at times negotiation-intensive’ (p. 4).^63^ One consortium shared their learning:

All collaborators should be aware of the fluid and the initially challenging processes that are normal for group development. Partners should allow sufficient time for complex and consultative decision making (p. 15)^42^

### Inequity and power imbalances

In all, 24 papers discussed inequity and power imbalances among partners, most often in terms of the inequitable division of resources, control and benefits. These were noted to have stemmed from pre-existing asymmetries between partners, as well as consortium design factors.^77 78^ Pre-existing asymmetries were reported to be based on differences in partners’ resources, income levels and expertise, with differences between North and South partners most often noted. These asymmetries predisposed consortia to power imbalances, exacerbated by consortium arrangements for access to funding, resource allocation and leadership.^77 78^ ‘Lopsided’ arrangements were reported to result in more-resourced partners taking up more conceptual roles and being perceived as capacity providers, and less-resourced partners becoming implementers and capacity receivers.^51 73 78–80^ Thus, unequal power relations are entrenched, and the ability of less-resourced partners to negotiate better terms undermined.

When the Northern partner serves as the primary grant recipient (and the Southern partner is subcontracted) a level of inequality is created that is difficult to overcome, no matter what provisions are made to make decisions equitably (p. 4)^77^

…it is too often assumed that the more developed nation has more to offer. This erroneous perspective is a fatal flaw in the development and progress of such collaborative efforts and is usually accountable for a number of failed attempts at collaboration due to its skewed balance of power (p. 101)^81^

“partners with less funding (almost entirely LMIC partners) confirmed that they felt as though they had less influence in decisions (p. 7)^60^

Power imbalances were not limited to North-South collaborations, but also encountered between ‘bigger’ and ‘smaller’ Southern partners.^48 68 78^ Openly acknowledging and discussing these issues were described as important in addressing this challenge in several papers^26 42 45 51 53^:

There are interests at stake among Southern universities just as there are among Northern universities… therefore power and interest dynamics are at play in South-South partnerships just as they are in North-South and North-North partnerships (p. 146)

Without honest exchange, and an acknowledgment of the differential power at work in seeking to resolve tensions in perspective, the notion of ‘equitable partnership’ was seen as illusory (p. 4)^26^

Others recommended negotiating and instituting consortium agreements and structures that promote power-sharing and equal division of resources, decision-making capacity and benefits,^19 63 77 81 82^ noting that these are not guarantees and sustained partner commitment to equal partnerships, mutual respect, trust, and reciprocity are still required.^48 50 52 55 73 77^

#### Lack of inclusion

Lack of inclusion of all partners especially during the early stages was raised as a concern, particularly of Southern consortium actors. In a Bangladesh–British partnership for instance, the project proposal was primarily developed by the Northern partner, resulting in implementation difficulties.^79^ Another author noted the following:

Many participants described their partnership experiences as more 'incorporation' than 'collaboration', having been provided little to no opportunity to participate in priority-setting or in leadership roles (p. 142)^51^

It was interesting to note that even in an LMIC-led consortium, decisions regarding a component being led by the high-income country partners were described as ‘top-down’ leading to some tension within the partnership.^42^ Across several papers, partner inclusion in all consortium processes, particularly in decision-making, was reported to engender ownership and commitment across both internal and external stakeholders.^10 52 63 81^ Inclusion of wider institutional actors, and being cognisant of host institutional leadership and structures when determining and executing consortium processes, was considered critical to HRCS success.^59 60^ In one consortium, the involvement of a wide range of stakeholders in conceptualising the HRCS project was seen to contribute to a ‘truly cooperative partnership based on trust and mutual respect’,^73^ while in others the lack of alignment with institutional agendas was considered detrimental.^43 60^

##### Leadership

Leadership was identified as a key attribute of successful consortium management in over a third of papers. It was deemed a major determinant of consortium success or failure,^49 83^ and its pivotal role was also demonstrated when some consortia faced leadership changes.^43 60 77^ As noted by an author,

A successful partnership requires strong leadership to make decisions, take appropriate risks, and solve problems (p. 6)^84^

In addition to providing direction and overseeing performance, vital aspects of leadership identified included demonstrating diplomacy and ensuring that partners are engaged throughout the consortium’s lifecycle.^45 62 63^ In one consortium, leaders’ commitment to inclusive partnership was considered instrumental in overcoming initial reservations of less-resourced partners in joining the consortium at all.^80^

### Effect of management processes and experiences on outcomes

Linkages between consortium management processes and programme outcomes were not clearly articulated, and only alluded to in a few recommendations made. Linkages made included observations that programme designs focusing on a wide range of human and infrastructural capacities^25 85 86^ across micro-, meso- and macro-levels^49 73 77 87^ produce more synergistic interactions and sustainable capacity. Acknowledging existing capacities of all partners and according mutual respect were noted to promote multidirectional capacity transfer,^29 49 81 88^ and correspondingly tailoring partners’ participation resulted in more contextually relevant and sustainable outcomes.^42 76 82 83^ The significance of consortium management in achieving research capacity strengthening outcomes is increasingly being acknowledged.^25 59 73^ Efficient management was named as one of four outputs in one consortium’s programme theory of change.^42^ Another paper identified the lack of management skills as a risk factor for consortia, criticising the reliance on the ‘learning-by-doing’ means of acquiring those skills which tends to happen late in consortia leaders’ careers.^82^ Some recognition of a more central capacity strengthening role of management activities was demonstrated in a few cases where partner interactions at both management and implementation levels were noted to generate exchange of knowledge and skills,^69^ and provide opportunities for mentoring and ‘behaviour modelling’.^73^ On the significance of these processes, one author pointed out:

What these [process] evaluation reports invariably facilitated was increased awareness of how underlying, often ignored or taken-for-granted processes influence project work and outcomes (p. 141)^25^

## Discussion

To the authors’ knowledge, no previous reviews have been conducted to ascertain the state of the evidence base for HRCS consortium management. This review seeks to provide a first step in assessing the consortium management publication landscape specifically in the HRCS domain and to draw attention to the need for purposeful HRCS-specific management science. We note that findings presented may not represent the entirety of HRCS consortia experiences. All but one paper reported successful collaborations, and discordant leader or partner perspectives were only reported in one case, indicating the possibility of publication and social desirability biases, respectively. Indeed, one participant disclosed their consortium’s deliberate decision not to report their ‘dirty laundry’ in a peer-reviewed publication.^48^ Thus, experiences of unsuccessful consortia may exist but are unpublished, and authors and study participants of selected papers may have been cautious in their publications and responses to avoid potential tensions and maintain relationships. Data from unpublished work or those published outside of peer-reviewed journals, or in languages other than English, or indexed in other databases, would have been excluded from this review. However, we used a systematic approach in carrying out the review ensuring a high level of rigour, and integrated diverse types of published literature to widen the range of included viewpoints.

The review indicates an increase in attention being given to HRCS consortium management-related issues in recent years. Yet, yearly publication outputs remain low, and the available evidence is weak both in terms of quantity and quality. Consortium management was not a clearly defined focus for most papers, and there was little coherence in its assessment across papers. The absence of LMIC authors in a significant proportion of publications also raises questions about the level of meaningful LMIC involvement and leadership in the LMIC-focused HRCS consortium management literature. Possible contributors to this authorship pattern include the dominance of high-income partners in consortium leadership, and broader structural and contextual factors which contribute to this imbalance such as resource and expertise constraints. Of note is that the nascent nature of the management-specific evidence reflects a similar trend in the broader HRCS literature, except that there is a better representation of LMIC authors in the latter.^34^ These imbalances and the factors contributing to them need to be addressed, with a particular emphasis on correcting the under-representation of LMIC perspectives in the available evidence.

Across the available evidence base, terms used for collaborations such as partnership, network and consortium are used inconsistently and interchangeably, a point also noted by others.^59 89^ Similarly, the concept of ‘(health) research capacity strengthening’ has been inconsistently applied across the broader HRCS literature.^34^ Thus, it is not entirely clear how an HRCS consortium might differ from a traditional health research consortium or how a consortium might differ from a partnership or network. Although not discussed in the literature, the lack of standard definitions and delineation of terminologies could lead to challenges with multiple perceptions of the nature and practices of a collaboration, as well as different partner expectations. Concerns about clarity in the use of terms contributed to efforts by Edwards *et al* 89 to develop a typology of international health partnerships to facilitate evaluations by positing a classification according to the level of impact (individual or organisational), capacity strengthening approach and the type of relationship between partners. Beyond ensuring the use of appropriate comparators in evaluation,^89^ characterising collaborations and being explicit about the attributes of the collaboration and degree of involvement, for instance, should promote consonance in partner thinking, approaches and expectations.

Our findings indicate greater emphasis on the relational aspects of management in the reviewed literature than on operational factors. Relational aspects such as relationship building, equity, power relations and leadership were identified as having the most influence on and requiring the greatest attention for successful HRCS consortium management. Though extensively mentioned, these elements were inadequately interrogated. It would be valuable to examine in more depth, for example, the different approaches to leadership (in theory and practice) and the sources and influences of power and power relations in the context of HRCS consortia. Operational aspects of management such as establishment processes, and governance structures and procedures, were given less attention. Given that relational and operational aspects of collaborations have been identified as interdependent elements of consortium management,^45 90 91^ it is unclear why the operational aspects are relatively neglected, and the interdependency and interplay between the two largely ignored. Only three papers hinted at any linkages.^45 77 78^ For example, Van der Veken *et al* 78 pointed out that inequity and power imbalances are as determined by consortium structures as they are by pre-existing contextual factors, and Vasquez *et al* 77 noted that formalised consortium structures are not sufficient in themselves in addressing power differentials and ensuring equity without commitment to the appropriate principles.

The lack of correlation between relational and operational elements in the literature is further evidenced in the linear nature of the partnership frameworks applied in the reviewed papers which rarely elicited the relational complexities inherent in consortium processes. Indeed, the importance of this interdependency is also recognised in business partnerships where emphasis is placed on going beyond formal governance structures to fostering collaborative relationships and behaviour.^92 93^ There is a growing recognition of the significance of this interplay in the health systems context where the need to equally pay attention to strengthening organisational hardware such as finances and technology, tangible software such as systems and procedures, and intangible software such as relationships and power has been emphasised.^94 95^ Thus, in future research, it will be worth examining how the relational and operational aspects enhance or hinder each other, and a first step will be to unpack and examine both the conceptual and practical content of each aspect particularly pertaining to the research capacity strengthening context. Exploring this interrelatedness will contribute to a more nuanced understanding of consortium management and contribute to the development of more holistic frameworks for guiding consortium operations and management.

Very little association has been made between HRCS consortium processes and capacity outcomes in the literature. There was almost no discussion in the reviewed literature on the ‘position’ of management in the HRCS effort and whether it merely supports a capacity development process or is a capacity development mechanism or target in its own right. This gap may be a result of the prevalent focus on HRCS activity outputs such as individuals trained and research conducted which are widely used as proxies for capacity,^96^ and the apparent prioritisation of technical research skills over managerial expertise. Though HRCS activities focus more on technical research tasks than non-technical relational skills, the emphasis in the HRCS consortium management literature is on the latter. This could be an indication that consortium processes may be segregated from the capacity strengthening process and only perceived as a means to an end. Though there is a growing recognition of the role of management in HRCS consortia, its handling in the available published literature is rudimentary. Even where management is explicitly named as an output, the focus remains on programme efficiency with management a facilitator of other programmatic outputs rather than a valuable capacity building output in itself.^42^ Besides, even programmes with explicit capacity strengthening strategies appear to be prioritising the ‘research’ over the ‘capacity’.^9^ In addition to ensuring HRCS programme models have ‘dedicated’ capacity strengthening foci,^9^ it is our view that the recognition and utilisation of management processes as capacity strengthening mechanisms in their own right are essential if research capacity goals are to be met. Considering the philosophy underpinning HRCS consortia,^1^ capacity development needs to permeate both processes and deliverables, and it is essential that both technical components and management approaches and processes adopted contribute to the capacity strengthening outcomes. As demonstrated by the UK Department for International Development’s example of impelling the incorporation of consortium management into programme theories of change,^30^ funders could play a key role in driving the prioritisation of consortium management and ensuring it receives adequate support (including funding) in its operationalisation and evaluation. This has been evidenced in the HRCS movement where funders such as the US National Institute of Health and the European Commission ensure that funding is committed to capacity building even in primarily research-oriented programmes.

## Conclusions

The consortium model has been widely adopted for strengthening health research capacity in LMICs. Yet, the evidence base to inform HRCS implementation is weak, and HRCS consortium actors lack the theoretical and empirical bases for framing their practice. From the limited evidence published to date, relational aspects of consortium management have been recognised as essential to HRCS programme success but not examined in depth. Operational processes have rarely been discussed, and it is unclear whether this is due to a lack of understanding or a lack of perceived importance. As a result, the interplay between operational and relational aspects of consortium management has not been well explored. The actual contribution of consortium management to HRCS outcomes is poorly documented, and the ‘position’ of management within the broader capacity strengthening agenda remains unclear. Considering the growing investments in consortia implementing the LMIC-focused HRCS agenda, it is essential to advance a corresponding consortium management framework to underpin the effort.

The proliferation of HRCS consortia provides opportunities for further research towards broadening the evidence base. The gaps identified in the literature highlight the need to pay more attention to both theoretical and empirical investigation of consortium management processes, influencing factors, and their role in attaining the capacity strengthening aims of consortia. Such research needs to aim for more conceptual depth, making use of robust study designs and adhering to research reporting requirements to overcome the quality problems identified. It is also essential to ensure definitional clarity and operational interpretation of key influencing factors such as equity, power and leadership particularly in the HRCS context, thus supporting appropriate translation into much-needed practical guidelines for funders and research practitioners. These may be useful initial steps in strengthening HRCS implementation science and boosting the evidence base needed for policy and practice.
