# Supplementary material for: Managing health research capacity strengthening consortia: a systematised review of the published literature
Source: BMJ Glob Health. 2019 Apr 14;4(2):e001318. doi: 10.1136/bmjgh-2018-001318 (PMC6509615; doi:10.1136/bmjgh-2018-001318)
Supplement: Supplementary data [file bmjgh-2018-001318supp001.pdf]

Table S1. Search terms and variants used in identification of papers

Goal	Intervention (variants combined by 'OR')	Mode (variants combined by 'OR')	Geographic location (variants combined by 'OR')
Health research capacity	Strengthening	Consorti*	LMIC
	Building	Collaborat*	Low* income
	Developing	Partner*	Low* and middle income
	Development	Network	South
		Alliance	Developing countr*
			Global
			Africa
			Asia
			Latin America
			Caribbean
			South America
			Pacific

Table S2. Supplementary data on included empirical papers on HRCS consortium management

Paper Reference	Study Design	Objective of paper	LMIC Geographical coverage[1]	Programme Components	Programme Focus	Study conducted by	MMAT Score[2]
Ager & Zarowsky 2015	Retrospective, summative, qualitative	Common challenges and their key drivers of HRCS	South, East & West Africa	RCS	Multiple	External	***
Anderson et al 2014	Retrospective, formative, qualitative	Evaluating the initial effects of a charter for collaboration	Africa - Ghana	Education RCS	Health education and research	Internal	Not scored
Birch et al 2013	Prospective, formative, qualitative	Self-evaluation of partnership using an identified assessment measure	Africa - Malawi	Clinical care Research	Nursing	Internal	**
Dean et al 2015	Retrospective, summative mixed methods	Lessons for establishing and maintaining successful research collaborations	West and East Africa	RCS	Health, agriculture, water and sanitation, biodiversity, and energy	External	**
Elmusharaf et al 2016	Retrospective, summative, qualitative	Achievements and outcomes of partnership	Africa - Sudan	RCS	Health systems	Internal and external	**
Farnman et al 2016	Retrospective, summative, qualitative	Successes, challenges and lessons learned	Africa - Uganda, South Africa, Tanzania Malawi<br><br>Asia - China, India, Oman, Vietnam	RCS	Health systems and services & Social determinants of health	Internal	***
Jentsch & Pilley 2003	Retrospective, summative, qualitative	Processes and dynamics within collaborations	Asia - Bangladesh & Thailand	Research RCS	Multiple	Internal	**
Larkan et al 2016	Retrospective, qualitative	Characteristics of successful research partnerships	Africa & Asia	Research RCS	Multiple	Internal	***
Mafigiri et al 2014	Prospective, formative, qualitative	Experiences, successes and challenges of collaboration	Africa - Uganda	Education RCS	Health education and research	External	**
Marjanovic et al 2013	Retrospective, formative, mixed methods	Experiences of consortia actors	Africa - East, West and South Africa	RCS	Multiple	External	Not scored
Mayhew et al 2008	Retrospective, summative, mixed methods	Successes and challenges, and lessons learned	Africa - South Africa<br>Asia - Thailand	RCS Research	Health Economics	External	*
Murphy et al 2015	Retrospective, qualitative	Research partnership experiences of stakeholders	Asia, Latin America and Africa	Research RCS	Multiple	Internal	**

Neuhann & Barteit 2017	Retrospective, summative, mixed methods	Outputs and outcomes including project's eventual failure, and lessons learnt	Africa - Malawi	Care Research RCS	Clinical care, health education & research	Internal	*
Redman-MacLaren et al 2012	Retrospective, summative, qualitative	Mutuality of research capacity strengthening	Pacific - Solomon Islands,	RCS	Nursing	Internal	****
Van der Veken et al 2017	Retrospective, formative, qualitative	Obstacles for southern institutions RCS & perceptions of southern researchers on capacity transfer	Africa - Burkina Faso, Benin, Senegal, Cote d'Ivoire, Guinea, Morocco, Tunisia, Algeria	RCS Research	Sexual and reproductive health	Internal	****
Varshney et al 2016	Retrospective, formative, qualitative	Understand challenges of collaborations, whether collaboration result in capacity building, and requirements for sustainable collaborations	Africa - South Africa<br>Asia - China, India, Oman, Vietnam	RCS	Social determinants of health	Not stated	***
Yarmoshuk et al 2018	Retrospective, summative, mixed methods	Describe partnerships characterized as higher-value for building the capacity of four universities and identify why they are so considered	Africa - Kenya, Tanzania	Education Research Practice RCS	Multiple	External	**
Yassi et al 2016	Retrospective, summative, qualitative	Determine the partnership model used, success factors and lessons learnt	Africa - South Africa	Practice Research RCS	Occupational health and infection control	Internal	****

(H)RCS – (Health) research capacity strengthening

[1]Specific countries or regions are listed if named in the publication

[2]MMAT scoring metrics: * = one criteria met (25%) to **** = all criteria met (100%); Not scored= did not fulfil screening criteria, thus not eligible for full appraisal

Table S3. Supplementary data on included commentary papers on HRCS consortium management

Paper Reference	Commentary informed by	Objective of paper	LMIC Geographical coverage[1]	Programme Components	Programme Focus
Airhihenbuwa et al 2011	Personal experience	Present partnership that led to training, and make recommendations for capacity building for health researchers in Africa	Africa - South Africa	RCS	HIV/AIDS
Ali et al 2012	Personal experience	Report on implementation, outputs and challenges of partnership	Asia - India	Research<br>RCS	Cancer
Asirwa et al 2016	Personal experience	Describe programme content, outcome measures and challenges	Africa - Kenya	Clinical care<br>Education<br>Research<br>RCS	Multiple health research areas
Atkins et al 2016	Personal experience	Describe the activities of two consortia	Africa - Uganda, South Africa, Tanzania Malawi<br><br>Asia - China, India, Oman, Vietnam)	RCS	Health systems and services & Social determinants of health
Breuer et al 2018	Personal experience	Reflect on the history, formation, challenges and achievements of the partnership	Africa - Ethiopia, South Africa, Uganda<br><br>Asia - India, Nepal	RCS<br>Research	Mental health
Cash-Gibson et al 2015	Personal experience	Description of a collaborative RCS project	Africa - Kenya, South Africa, Tanzania<br><br>Latin America & Caribbean - Brazil, Mexico, Colombia	RCS	Social determinants of health
Chandiwana & Ornbjerg 2003	Review	Discuss lessons in North-South and South-South cooperation	Zimbabwe, Southern Africa	RCS	Public health research
Dalmar et al 2017	Personal experience	Outline reasons and motivations for re-launching research cooperation programme	Africa - Somalia	RCS	Health research

de-Graft Aikins et al 2012	Personal experience	Review the partnership's achievements and challenges	Africa - Ghana, Burkina Faso, Nigeria, Kenya, Cameroun, South Africa<br><br>Asia - Malaysia	Research<br>RCS	Chronic diseases
Eckerle et al 2017	Personal experience	Describe evolution of novel global health partnership	Africa - Malawi	Clinical care<br>Education<br>Research<br>RCS	Paediatric emergencies
Ezeh et al 2010	Personal experience	Describe the consortium	Africa - Kenya, South Africa, Tanzania, Uganda, Nigeria, Malawi, Rwanda	RCS	Population and public health
Fischer et al 2017	Personal experience	Describe steps taken and key factors in establishing a successful collaborative consortium, and challenges	Africa - Nigeria	Research<br>Clinical care<br>RCS	Cancers
Greenwood et al 2012	Personal experience	Key lessons learnt	Africa - Malawi, Tanzania, Gambia, Ghana	RCS<br>Research	Malaria
Greenwood et al 2018	Personal experience	Describe programme activities and lessons learnt	Africa - Senegal, Malawi, Tanzania, Uganda, Ghana	RCS	Malaria
Gureje et al 2018	Personal experience	Describe the partnership, programme components, and challenges	Africa - Nigeria, South Africa, Ghana, Kenya and Liberia	Research<br>RCS	Mental health
Kaddumukasa et al 2014	Personal experience	Describe partnership initiative	Africa - Uganda	RCS<br>Education	Neurology
Kutcher et al 2010	Personal experience	Describe the process and activities of a partnership	Cuba, Latin America & Caribbean	Research<br>RCS	Mental health
MacLaren et al 2015	Personal experience	Provide an example of a RCS model	Pacific - Solomon Islands	RCS	Nursing
Mathai et al 2018	Personal experience	Describe a South-North collaboration	Africa - Kenya	RCS<br>Research	Mental health
Miiro et al 2013	Personal experience	Outline the initial experiences of the merits, outputs and lessons learnt in 4 networks	West, East, Central and Southern Africa	Research<br>RCS	HIV/, TB, Malaria and NTDs

Miranda et al 2018	Personal experience	Explore features of existing partnerships	Latin America & Caribbean	Research RCS	Non-communicable diseases
Nakabugo et al 2010	Personal experience	Illustrate potential mutual benefits from partnerships, challenges and strategies for achieving a mutual and sustainable partnership	Africa - Malawi, Mozambique, Tanzania, Uganda	RCS	Health and education
Noormahomed et al 2017	Personal experience	To describe programme outcomes, compare main features with traditional collaboration models; and describe sustainability strategies	Africa - Mozambique	Education RCS	Health education and research
O'Connor et al 2016	Personal experience	Present results and lessons learnt	Africa - Malawi	Education Research RCS	Nursing informatics
Ogden & Porter 2000	Personal experience	Recount the unfolding of two related international research collaborations	Asia - India	Research RCS	Tuberculosis
Onokerhoraye & Maticka-Tyndale 2012	Personal experience	Examine the experience of the partnership and outlining successes and challenges	Africa - Nigeria	Research RCS, Knowledge translation	HIV/AID
O'Sullivan et al 2017	Personal experience	Describe the approach used to develop a collaborative and sustainable partnership, and initial project outcomes	Africa - Uganda	Education RCS	Physiotherapy
Pinto et al 2012	Personal experience	Using the International Participatory Research Framework (IPRF) to build a partnership	Latin America & Caribbean - Brazil	Research RCS	HIV/AIDS
Reddy et al 2002	Personal experience	Discussion of challenges and opportunities for capacity building and collaboration strategies	Africa - South Africa	RCS	HIV/AIDS
Sanchez at al 2013	Personal experience	Describe the implementation, challenges, and lessons learned of an RCS project	Latin America & Caribbean - Honduras	RCS	Infectious diseases
Semrau et al 2018	Personal experience	Discuss programme approaches and outputs	Africa - Ethiopia, Nigeria, South Africa and Uganda<br><br>Asia - India, Nepal	Clinical care RCS	Mental health

Silva et al 1994	Personal experience	Review the results of the programme from the angle of cross-fertilization of disciplines through their collaboration	Asia – Sri Lanka	RCS<br>Research	Health social science
Spiegel et al 2006	Personal experience	Summarize how collaboration was pursued, identify outputs, and highlight mutual benefits gained	Latin America & Caribbean - Cuba	Education<br>Research<br>RCS	Environmental Health
Stillman et al 2006	Personal experience	Describe the approach, main outcomes and challenges of a partnership	Asia - China, Latin America & Caribbean - Brazil, Mexico	RCS<br>Research	Tobacco control
Tierney et al 2013	Personal experience	Describe partnership, challenges and value created	Africa - Kenya	Clinical care<br>Education<br>Research<br>RCS	Multiple health research areas
Van Teijlingen et al 2018	Personal experience	Highlight reasons for, considerations, and procedures around development of partnerships and key challenges	Asia = Nepal	Education<br>RCS<br>Research	Not stated
Vasques et al 2013	Personal experience	Highlight major challenges to the HRCS enterprise and make recommendations	Asia - Vietnam	RCS	HIV/AIDS

(H)RCS - (Health) research capacity strengthening

[1]Specific countries or regions are listed if named in the publication
